# Laparoscopic Salpingectomy for Isolated Fallopian Tube Torsion in the Third Trimester

**DOI:** 10.1155/2012/239352

**Published:** 2012-09-18

**Authors:** R. P. Duncan, M. M. Shah

**Affiliations:** Department of Obstetrics and Gynaecology, Gosford Hospital, Gosford, NSW 2250, Australia

## Abstract

Isolated tubal torsion is a rare event. The clinical presentation is often nonspecific and the diagnosis is difficult, especially in the gravida abdomen. If left untreated, torsion can result in premature labour and foetal loss, as well as maternal morbidity. Here we present a case of isolated tubal torsion in a primigravida occurring in her third trimester and subsequent successful laparoscopic salpingectomy, rather than laparotomy. We discuss some of the diagnostic difficulties faced and approached to surgery as well as a brief review of the literature. In our case the women went on to successfully complete her pregnancy with no further complications.

## 1. Introduction

Isolated tubal torsion is a rare event. The clinical presentation is often nonspecific and the diagnosis, difficult, especially in the gravida abdomen. If left untreated, torsion can result in premature labour and foetal loss, as well as maternal morbidity. Here we present a case of isolated tubal torsion in a primigravida occurring in her third trimester and subsequent successful laparoscopic salpingectomy, rather than laparotomy.

## 2. Case Report

A 28-year-old female, G1P0 30^+3^/40 gestation presented with sudden onset severe right-sided abdominal pain described as an intermittent sharp stabbing pain radiating to her right flank. Her pain was associated with three episodes of vomiting but no diarrhoea, constipation, or fevers. On examination her vital signs were unremarkable; temperature of 37.2°C, pulse 80, blood pressure 134/70, respiratory rate 14 and saturating at 100% onroom air. Her abdomen was diffusely tender in all quadrants with increased tenderness of the right flank and epigastric region. Systems review and examination were otherwise unremarkable. CTG was normal and reactive with good foetal movements. On admission she had a white cell count of 11.9 × 10^9^/L  H, lipase 179 U/L, and a raised CRP of 9.3 mg/L H. Serum electrolytes, liver function tests, and full blood count were otherwise normal. Her urine dipstick showed trace protein and leukocytes and subsequently grew no bacteria.

The patient had no history of gallstones, renal calculi, or previous UTI's and was otherwise well with no medical issues of note. On her dating scan at 7^+3^/40 gestation a right adnexal cyst of uncertain origin, measuring 35 mm × 34 mm × 35 mm, was noted. The patient had conceived naturally and her antenatal history was largely unremarkable aside from an increased risk of trisomy 13 and 18 at her nuchal scan which was later confirmed to be normal via chorionic villus sampling. She was not on any regular medications aside from the pregnancy multivitamin Elevit.

As an inpatient an ultrasound was performed which revealed a single live intrauterine pregnancy consistent with 30-week gestation. Kidneys and renal tract were normal. The appendix was not visualised but a 47 mm oblong cyst was seen in the right adnexa. The left adnexa and ovary were reported as normal. Her pain began to settle with 15 mg of morphine and 10 mg of Buscopan. She was given two doses of 11.4 mg Celestone IM 12 hours apart and kept nil by mouth overnight.

The following day her symptoms remained unresolved. The patient continued to have intermittent severe pain and her CRP and neutrophilia were increasing. The decision was made to proceed to surgery. The risk of premature labour secondary to intra-abdominal infection was a deciding factor. At this point her primary differential diagnosis was that the previously identified adnexal mass was undergoing torsion with subsequent necrosis and infection. Other differentials included tuboovarian torsion, atypical appendicitis, gall bladder, and hepatic pathologies. The patient was consented for an exploratory laparoscopy for confirmation of the diagnosis and subsequent laparotomy.

Intraoperatively, atrochar was inserted into the epigastrium, pneumoperitoneum achieved, and camera inserted to view the mass. A torted right hydrosalpinx was seen with an associated fimbrial cyst. The left ovary and tube appeared grossly normal. The cyst was approximately 50 mm and haemorrhagic but was otherwise simple in appearance. The mass was deemed removable by laparoscopy and two further ports were inserted as per traditional laparoscopic cholecystectomy positioning, to accommodate the gravid uterus. The torted hydrosalpinx was detorted; however, the fallopian tube had been compromised and it was necessary to proceed with removal. A right salpingectomy was performed. The tube was ligated adjacent to the uterus using 2xPDS and divided. It was placed in an endocatch bag, drained, and removed. The port sites were closed with subcuticular sutures. Prophylactic tocolysis was not used and uterine irritability did not develop postoperatively. Anatomical Pathology confirmed the diagnosis of a simple, uniloculated fimbrial cyst (44 × 22 × 10 mm) and ischemic fallopian tube which was otherwise histologically normal.

The patient had an uncomplicated postoperative recovery and continued the pregnancy with no further issues. She presented at 38^+3^/40 gestation with spontaneous rupture of membranes and went on to have an emergency caesarean section, unrelated to her previous operation, for foetal distress. Postpartum recovery was unremarkable.

## 3. Discussion

The occurrence of isolated tubal torsion is a rare event, especially in pregnancy [1]. Diagnosis is difficult due to the nonspecific nature of the presentation. Patients often present with unilateral abdominal pain associated with nausea and sometimes fever. In most presentations the clinician will have a list of differential diagnoses which cannot be excluded until the time of surgery. In pregnant women these include appendicitis, biliary pathologies, pelvic inflammatory disease, leiomyoma-related events, cyst accidents, diverticular disease, maternal hydronephrosis, placental abruption, and ovarian and fallopian tube torsion. Pre-existing risk factors for torsion are common and include tubal pathologies such as paratubal cysts, hydrosalpinx, neoplasm, congenital anomalies, and tubal ligation devices. Other risk factors include ovarian masses, ovarian hyper-stimulation, infection, ectopic pregnancies, extrinsic lesions such as adhesions and endometriosis in addition to trauma [2].

In this case our patient had a known likely tuboovarian cyst identified prior to pregnancy which measured 35 mm^3^ at the time of her dating ultrasound. Given her history and subsequent ultrasound showing a new oblong mass in the adnexa, the diagnosis of a tubal torsion secondary to a pre-existing cyst was strongly suspected prior to surgery. Adnexal masses are well known to cause torsions during pregnancy. A previous study of adnexal masses in pregnancy found that masses between 60–80 mm were most likely to cause torsion and 94% of torsions occurred before 20-week gestation [3].

With the increased availability of more advanced imaging methods, there is a trend towards the use of Magnetic Resonance Imaging (MRI) in helping with diagnosis prior to surgery in pregnancy. In our case ultrasound was the only imaging modality used, as the patient's pain was quite severe and warranted immediate surgical intervention. However, in cases where the pain experienced is milder, and time to surgery is not as critical further investigation can be performed. MRI is emerging as an alternative option to diagnostic surgery and is becoming more widely used in larger centres where access to this modality is more readily available for acute presentations [4].

Unless imaging is able to conclusively exclude the need, early surgical intervention is recommended for pregnant women presenting with an acute abdomen irrespective of their gestation. This is due to the dire complications that can result from a conservative “wait and see approach”. Premature labour is far more likely, and the risk of maternal morbidity and mortality higher, if infection of the abdominal cavity is allowed to occur [5]. A torted adnexal mass undergoing necrosis has potential to cause serious infection similar to that of a perforated appendicitis [5]. Therefore in pregnant women the risk of complications associated with an acute abdomen outweighs the risks associated with abdominal surgery. Additionally, early intervention can sometimes salvage torted tissue by detorsion if it is performed in time [6]. In our case the patient had essentially developed an acute surgical abdomen with deteriorating inflammatory markers, and it was considered that the risks of surgical intervention were outweighed by the risks of conservative management. 

The technical approach in surgical intervention is also an important consideration with the gravid abdomen. Depending on the pathology suspected and the skill of the operator, either laparoscopic or open approach (i.e., laparotomy) can be used with similar overall outcomes for maternal and foetal wellbeing [7]. In cases where a laparoscopic approach is adopted it is particularly important to carefully plan port site placement, especially in the third trimester. In our case we opted for initial diagnostic laparoscopic investigation of the pathology with the intent to use the laparoscope for identification of the appropriate site for subsequent laparotomy incision. However, intraoperatively it was decided that a wholly laparoscopic approach was safe and appropriate. The advantage of performing laparoscopic surgery in pregnancy is generally accepted to be shorter hospital stays and lower rates of premature labour. This has been demonstrated by a recent study by Walsh et al. which focused on appendectomies in pregnancy and found a decreased rate of preterm delivery with a laparoscopic approach (2.1% versus 8.1%) in a large meta-analysis of 637 patients [8]. This was independent of the use of tocolysis which did not significantly reduce the rate of preterm labour. Interestingly, this paper also reports an increased rate of foetal loss with laparoscopic surgery versus laparotomy (5.6% versus 3.1%). However, the majority of the cases resulting in foetal loss were of complicated appendicitis (defined as evidence of perforation, appendiceal abscesses, or generalised peritonitis). The conclusion which could be drawn from this study is that while laparoscopy is the preferred approach and has less overall complication, conversion to laparotomy is warranted if during the laparoscopy the pathology is deemed “complicated” in order to avoid the reported increased rate of foetal loss, especially in the first and second trimester.

This paper has described a case of isolated tubal torsion in the third trimester of pregnancy successfully treated by laparoscopic surgery alone with subsequent completion of the pregnancy. Aggressive management including early surgical invention is indicated in such cases to prevent foetal and maternal morbidity and mortality.
